# Transition to Motherhood of Mothers Receiving Continuity of Child-Rearing Support

**DOI:** 10.3390/ijerph19148440

**Published:** 2022-07-11

**Authors:** Mai Itai, Shizuka Harada, Ryoko Nakazato, Shinobu Sakurai

**Affiliations:** Faculty of Health Care and Nursing, Juntendo University, Urayasu 279-0023, Chiba, Japan; sharada@juntendo.ac.jp (S.H.); r-nakazato@juntendo.ac.jp (R.N.); ssakura@juntendo.ac.jp (S.S.)

**Keywords:** transition, mothers, parenting, child rearing, support

## Abstract

Clarifying the transition to motherhood based on the experience of mothers receiving continuity of child-rearing support is expected to promote the transition, and enhance nursing support. This study clarifies the transition process by which mothers recognize and adapt to new roles with continuity of child-rearing support in Japan. Semi-structured interviews were conducted with 13 mothers who received continuity of child-rearing support. Directed content analysis based on Meleis’s Transition Theory was used to analyze the results and define a framework for analyzing the transition. A theoretical framework was identified based on the Transition Theory themes. Engagement in terms of proactive involvement in pregnancy, childbirth, and child-rearing was a transition property. Maintaining the mother’s well-being, building new relationships and connections, and reflecting on child-rearing in the community were transition conditions. For patterns of response, the process indicators comprised having a sense of connection with the community and reflecting on one’s child-rearing objectively. Increased readiness for child-rearing and identity re-shaping were outcome indicators. The results suggest that it is important to enhance mothers’ readiness for child-rearing for transition to motherhood, and to promote the reshaping of their identities through continuity of support from pregnancy to postpartum child-rearing.

## 1. Introduction

Currently, the environment in Japan surrounding parents and children is completely different from that of the past, owing to the declining birthrate, nuclearization of family, and urbanization [1]. With the nuclearization of families and women’s increased participation in the workforce [2,3], mothers do not have enough time or opportunity to ask for help or consult someone about child-rearing, and are prone to isolation. Mothers with higher child-rearing anxiety have been found to have less support from their husbands and less social support [4]. Under these circumstances, mothers struggle with the unfamiliarity of child-rearing, and are burdened and stressed [5].

Pregnancy and childbirth are events that lead to a transition process; transitions are periods between fairly stable states [6]. Women experiencing physical and mental changes such as pregnancy and childbirth undergo a transitional process of recognizing and adapting to new roles as mothers. In order to support this unstable and stressful transition process, child-rearing support is provided, mainly by municipalities and communities, in Japan. Since the nuclearization of families has weakened family ties and cooperative relationships, the need for support in the transition to motherhood and child-rearing from municipalities and communities is increasing [7]. However, continuity of child-rearing support for this transition is not easy. In addition to moving in and out due to urbanization, there are institutional issues as well. In Japan, pregnant women with or without physical risks can receive health checkups as per the Maternal and Child Health Law, and parenting support after birth is mainly carried out by municipality- and community-based public health centers. The mothers receive support from different sectors: primarily from medical doctors during pregnancy, and public health nurses in a community center postpartum. Information-sharing between these supporters is an important issue from the viewpoint of personal information protection, especially in contexts such as child abuse. However, continuity of support from pregnancy to parenting is important for all mothers and children. The importance of creating integrated services and seamless child-rearing support to enhance postpartum health and wellness has been suggested [8].

Japan is trying to change the child-rearing support system to “continuity of support” in order to improve maternal and child health. The Ministry of Health, Labor and Welfare implemented a comprehensive pregnancy and childbirth support model project in 2014 with the aim of strengthening continuity of child-rearing support at the local level. One of these model projects was carried out in a city adjacent to the capital, with a population of approximately 160,000. In this city, more than 90% of child-rearing families were nuclear families, and it had regional characteristics with many families moving in and out. Mothers tended to have a strong sense of burden and isolation in raising their children in this city. As a form of “continuity of child-rearing support”, the city launched the Children’s Project, referencing Neuvola of Finland. In addition to the conventional child-rearing support and public health service, the Children’s Project created childcare plans and distributed child-rearing support gifts for all parents. The childcare plan was prepared by the city’s certified and hired childcare support advisers, who considered the concerns and anxieties of parents and their childcare needs. Childcare support advisers are those who are interested in childcare support in the community, and voluntarily participate in training sessions conducted by the city. Many of them have experience with child-rearing. The function of these community childcare support advisers is to help to provide continuity of care to the mothers. Public health nurses work with childcare support advisers to support mothers and children. Public health nurses, as professionals, are especially involved in cases of physical and mental high risk, and are responsible for overall management. The childcare plan supported parents from the time of pregnancy, through childbirth, until approximately the second birthday of the child. The childcare plans are created at least three times. Through the creation of a child-rearing plan, information is provided on planning out the pregnancy period, utilizing child-rearing support services, setting goals for childbirth and child-rearing, and consulting regarding problems. This childcare plan allows mothers and their supporters to share goals and provide repeated and continuous support toward the same goals. Childcare support advisers are available for pregnant women and families raising children, and act as a bridge to public health nurses when necessary. There is a place in the community health center where childcare support advisers and public health nurses create childcare plans, listen to the concerns, and introduce some social resources. Mothers can visit the place when they want to be supported from pregnancy to birth and beyond. In the “distribution of child-rearing support gifts”, diaper bags, baby clothes, and other items necessary for child-rearing, as well as vouchers are distributed. This is not only financial support, but also educational support, in that it introduces necessary items for raising children. By providing such support continuously at important milestones during the development of mothers and children, a smooth transition to motherhood can be supported.

Since most of the existing research on the transition to motherhood has studied white, married, and middle-class individuals, it is necessary to expand it to a wider range of socioeconomic classes and to more culturally diverse participants as they transition into motherhood [9]. Recently, there have been studies targeting adolescent mothers [10], blind women [11], women in wheelchairs [12], and women with infants with special care needs [13]. A review has shown that the transition to motherhood includes two inherent processes: (1) engagement and (2) growth and transformation [9]. This review suggests that there are important supports for maternal transition, such as prenatal discussion of realistic expectations for the transitional period, ongoing support through the first 6 months postpartum, and the use of role models [8]. Continuity of child-rearing support has the potential to promote engagement, growth, and transformation, and facilitate the transition of mothers [8,9]. Few studies have focused on the transition to motherhood from the perspective of mothers raising children while receiving continuity of child-rearing support, or have examined the support required for promoting the transition of mothers.

The purpose of this study was to clarify the transition process in which mothers in Japan recognize and adapt to their new roles. By clarifying their transition to motherhood, we can understand the factors that promote transition and provide nursing support, even in difficult situations surrounding mothers and children.

## 2. Methods

### 2.1. Research Design

A descriptive qualitative study design was used. The qualitative descriptive approach results provide an understanding of the selected phenomenon. 

### 2.2. Research Participants

The research participants from convenience sampling consisted of 13 mothers: (1) whose children were born between May and June 2015, (2) who resided in the aforementioned city in Japan with a population of approximately 160,000, and (3) who had developed a parenting childcare plan and could share their past and current child-rearing experiences. Since the purpose of this study was to clarify the transition of mothers raising children while receiving “continuity of child-rearing support”, which is a pioneering initiative in Japan, the participants were residents of the city that implemented the Children’s Project, who could offer research cooperation. All participants were Japanese women who could cooperate with the interview in Japanese.

### 2.3. Data Collection

Semi-structured interviews were conducted with mothers about their experiences of parenting while receiving continuity of child-rearing support from the municipality during pregnancy till after giving birth. Data were collected between December 2016 and February 2017 at a health center or at a location designated by the participants. The content of the interviews was initially determined by discussion among the researchers, and included concerns and difficulties in raising children and dealing with them, as well as experiences and thoughts about the continuity of child-rearing support and the child-rearing environment. Recruitment at a public health center involved distributing handouts and providing oral explanations of the study at the time of the public health nurse’s consultations. The interviews were conducted for about 45 to 60 min each with the consent of the participants, and were recorded with an IC recorder.

### 2.4. Analysis Method

This study conducted directed content analysis [14] based on the Transition Theory. Transition denotes a change in health status, role relationships, expectations, or abilities, and nurses deal with people who are experiencing, anticipating, or completing the act of transition [15]. A central concept in nursing defined a framework for analyzing transitions, identifying their components, and clarifying and reflecting the relationships between these components [16]. The emerging middle-range theory of transitions consists of the nature of transitions, transition conditions, patterns of response and nursing therapeutics [6]. This study focused on continuity of child-rearing support as therapeutics and transition to motherhood. Therefore, based on the framework and concepts of Transition Theory by Meleis, we decided to identify the components of the above.

Verbatim transcripts of the recorded interviews were created, and the data were sectioned and given labels. The similarity of labels was examined and coded by semantic content. Key concepts and variables were identified as the first coding categories based on Transition Theory. Key concepts and variables identified from existing theories were structured and compared to the codes and categories extracted from the interviews. Codes and categories that did not match the concepts identified from the Transition Theory were examined to determine whether they should be represented as new categories or subcategories of existing codes. To increase the reliability and validity of the analysis, the analysis was conducted by several researchers with experience in qualitative research independently, and there were repeated discussions among the researchers. In cases of disagreement of the theme, analysis and discussion were repeated until the researchers agreed. The reporting was carried out according to the Consolidated Criteria for Reporting Qualitative Research (COREQ) [17].

### 2.5. Ethical Considerations

Before the study was conducted, a document was used to explain, orally and in writing, the details of the study, the storage and handling of data, any disadvantages to individuals arising from this research, matters concerning withdrawal of consent, protection of personal privacy, disclosure of information, publication of research results, and the person responsible for the research and contact information. Consent for research cooperation and interview recording was obtained using the consent form.

## 3. Results

### 3.1. Outline of the Participants

Participants’ age ranged from 28 to 42 years (M age = 35.4 years), and they had one to two children. Out of the 13 participants, 7 (53.8%) were employed (Table 1). The total fertility rate in Japan in 2019 was 1.36 [18], and the number of children per mother in this study was similar to the national average.

### 3.2. Transition to Motherhood with Continuity of Child-Rearing Support

**Figure 1:**
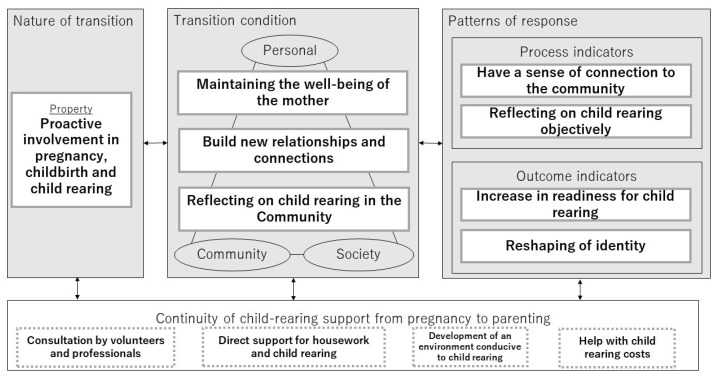
Theoretical framework of the transition to motherhood with child-rearing support continuously.

The results of a directed content analysis based on Meleis’s Transition Theory revealed a theoretical framework of transition to motherhood with continuity of child-rearing support (Figure 1). The results are described according to the four themes of the framework.

#### 3.2.1. Nature of Transition

In this study, we clarified the engagement in pregnancy, childbirth, and child-rearing as a “property” of “nature of transition” in the transition to motherhood of mothers receiving continuity of child-rearing support. Mothers were proactively involved in pregnancy, childbirth, and child-rearing as they wished to become pregnant, prepare for child-rearing, and adjust to the living environment and their social role. Constructive activity corrections, such as changing their work style, adjusting social role, and relocation due to lifestyle changes, were seen from pregnancy to childbirth. Mothers reported, “Find and identify the services that I can use when I need help raising my child”, and “Moved to a larger house after the birth”.

#### 3.2.2. Transition Conditions

Maintaining the mother’s well-being, building new relationships and connections, and reflecting on child-rearing in the community were identified as “transition conditions”. 

The mothers maintained their well-being by coping with the physical and mental changes associated with pregnancy and childbirth. In addition, mothers strengthened relationships with their families, including their husbands and parents, and obtained support from them. They also built relationships with others going through the same transition using connections and through mutually helping each other. Furthermore, the mothers chose their living environment by adjusting their physical distance from their parents, such as moving closer or farther to their parents’ home, or by making independent choices about where to live, such as moving due to work or lifestyle changes. Some mothers said it was reassuring to have their parents’ home close by, whereas others said that the distance kept them from interfering excessively with each other. Through these actions, mothers established new relationships and connections. In addition, the mothers understood, learned, and thought about how to utilize the support—at times, choosing to not use it. In the process of reflecting on child-rearing in the community, the mothers evaluated child-rearing support services in retrospect, and were satisfied with the child-rearing environment. Mothers mentioned their satisfaction with childcare support services, trust in supporters, and the convenience in using public facilities such as community health centers, parks, etc.

#### 3.2.3. Patterns of Response

The “process indicators” included having a sense of connection with the community and objectively reflecting on one’s child-rearing. By having a sense of familiarity with the area and the local community, the mothers were able to recognize the connection with the community. Mothers said, “Through my child, I gained more friends than I had expected”, “There are a lots of events related to children and not isolated”, and “The child-rearing support gifts we received from the city vary from year to year, so when we had the same items, we knew our children are the same age and felt closer to them”. Through these experiences, the mothers became attached and felt connected to the community. By comparing, reflecting, and looking forward to child-rearing with the community, mothers were able to objectively reflect on their child-rearing experience. They compared their children with their siblings and other children, the differences in childcare practices over time, and the childcare services they had received in their previous residence. Through these comparisons, they perceived their characteristics, anxieties, and hesitations, and reflected upon their feelings about child-rearing. In recognizing the various challenges, they were able to have an objective view of their child-rearing by envisioning the near future and looking ahead.

The “outcome indicators” comprised increased readiness for child-rearing and identity re-shaping. The literacy about child-rearing improved through continuity of child-rearing support. By actively collecting and utilizing information and using the services, the participants learned how to raise their children, and did so in their own way, thereby improving their readiness to raise children. Through the creation of a childcare plan, they set their own goals during pregnancy and child-rearing, and proactively developed their careers, including a sense of roles other than motherhood and balancing work. Through positive acceptance of their childcare and changes in their views on childcare, they gave meaning to child-rearing and reshaped their identities.

#### 3.2.4. Therapeutics: Continuity of Child-Rearing Support from Pregnancy to Parenting

The therapeutic that facilitated these transition processes was continuity of child-rearing support from pregnancy to the child-rearing period. It included the provision of information on childbirth and child-rearing, instructions on how to obtain information independently, encouragement for self-reflection, and consultation with childcare support advisers and public health nurses who were available to provide a safe space to talk.

In their consultations with mothers, the childcare support advisers and public health nurses assessed not only the current problems, but also anticipated situations and difficulties, and provided support by conveying information in advance. In addition to receiving information, mothers were also taught how to obtain information independently. One mother reported, “This was the first opportunity for me to look into various things about children and child-rearing”. Furthermore, mothers were encouraged to set their own goals for raising their children through the creation of childcare plans. Another mother revealed, “Being asked, ‘What do you want your child to be when they grow up?’ was a good opportunity for me to reflect”. A participant also shared, “Just having someone listen to me and tell me it’s going to be okay made a difference in how I feel”. The childcare support advisers and public health nurses were perceived as people with whom they could talk without reservations.

There was direct support for housework and child-rearing, such as the use of child-rearing helpers and temporary childcare services. In addition to tangible support, the city also provided support in the form of the development of an environment conducive to child-rearing, such as providing a space where people could talk in a relaxed manner, and facilities and events to which people could go out with their children. Assistance with child-rearing costs included financial support (vouchers) and the provision of materials required in child-rearing. Support was also provided to families with financial problems, which not only included financial support, but also helping them recognize their connections in the community. One mother reported, “I find it easier to talk to other mothers when I make my child wear matching clothes and bags given by the city”.

## 4. Discussion

Some studies have used forms of the word “engage” to describe maternal transition [19,20,21]. As a “nature of transition”, the “property” of proactive involvement in pregnancy, childbirth, and child-rearing was identified. The participants in this study received continuity of child-rearing support, which enhanced this property. Specifically, the support provided multiple opportunities for dialog with childcare support advisers and public health nurses, and the “property” of proactive involvement in pregnancy, childbirth, and child-rearing was strengthened by the provision of informative health counseling and the encouragement of self-reflection. Previous studies have revealed that the sense of security during the first week after birth is statistically significantly greater in mothers whose husbands actively participated in psychophysical preparation for childbirth together with them [22]. In addition to family participation in child-rearing, having someone other than family members to consult with about child-rearing and to turn to in times of need can lead to a sense of security, and may promote proactive involvement in child-rearing. In addition, it was suggested that support that encourages opportunities for mothers to reflect upon their experiences during the busy parenting period is an important aid in the transition to motherhood. The creation of a childcare plan as a part of the continuity of child-rearing support was not only significant for providing information on child-rearing, but was also expected to function as a tool for encouraging mothers to actively “engage” in child-rearing.

As a transition condition, it was important to maintain the mothers’ well-being. Pregnancy is a complex and vulnerable period that presents a number of challenges to women, including the development of postpartum psychiatric disorders [23]. Physical problems have been shown to affect adolescent mothers’ experiences of transitioning to motherhood [10]. It is important for women to cope with the physical and mental changes that accompany pregnancy and childbirth, and maintain their well-being. Particularly after childbirth, women take on many tasks, including housework, child-rearing, and social roles related to work, leading to fatigue and stress. To reduce the burden of child-rearing and to maintain the mother’s well-being, it is important to establish social resources and support systems that directly support housework and child-rearing, such as child-rearing helpers and temporary childcare services. In addition, it was suggested that the provision of child-rearing goods is not only a form of financial support, but also supports the recognition of their connection with the community. Child-rearing goods were thought to be important for mothers to know what they needed to raise their children, and mothers felt a sense of closeness with other mothers through the matching child-rearing goods provided by the city. The mothers recognized the connection with the community through child-rearing support and built relationships with other mothers. It was important for them to reflect on child-rearing in the community and build new relationships and connections to get the sense that child-rearing was not only an individual effort, but was also positioned in the community and society through child-rearing support. Several previous studies on transition to motherhood have referred to partner and family support and the changed relationships with them [9,11,12,13], with similar results to this study, which showed that building new relationships and connections was key. Conversely, a new finding of reflecting on child-rearing in the community was clarified as a transition condition, not only the relationships with partners and families. The mothers received psychological support from the pregnancy to the child-rearing period continuously. At the same time, this promoted a shift in the perspective of mothers from the individual to the community and society in order to adjust to these transition conditions.

To prevent isolation, it is necessary to socialize during child-rearing. Mothers’ and local residents’ awareness of the elements of the social constitution was identified as a factor that influences willingness and positive attitudes toward cooperative child-rearing; in particular, connections and social interaction were shown to have primary influences [24]. The study participants consulted with childcare support advisers and public health nurses who they could talk to safely, and actively used local information and services to visit facilities and events to which they could go out with their children. Through interventions using peer facilitators, there were positive trends in community service use, particularly postnatally [25]. We believe that the behavior and mindset of mothers who are not alone in child-rearing, who seek help in times of need, and who proactively use local services are important elements of the socialization of child-rearing. In addition, as a result of this study, the mothers’ sense of connectedness to the community and the objective view of their child-rearing through continuity of child-rearing support increased their readiness for child-rearing and reshaped their identities. We believe that having a sense of attachment to the community and recognizing one’s connection to it through child-rearing prevents the isolation of mothers and children and reduces the mental burden on mothers. Previous research has suggested that women who experience perinatal mental health difficulties value specialist perinatal expertise, but that general, non-perinatal teams may also have advantages for some [26]. We believe that it is possible to strengthen the direct connection with the mother through the active involvement of child-rearing supporters, as well as promote connections indirectly. For example, it is possible to introduce local resources when necessary and create opportunities for mothers to connect with each other and with the local community, thereby encouraging awareness of the connection with the community and society.

Birth rates are declining year-by-year, and there are fewer opportunities to raise children. It is not easy to learn about pregnancy, childbirth, and child-rearing in our daily life. In this continuity of child-rearing support from pregnancy to parenting, childcare support advisers and public health nurses provided multiple and continuous opportunities for giving information and counseling through making childcare plans. Continuity of support is one of the issues, because mothers receive support from different sectors before and after they give birth in Japan. However, our results suggested that continuity of child-rearing support can help the transition to motherhood, e.g., in Finland, regular visits to Neuvola during pregnancy, postpartum, and until the child enters elementary school to receive professional’s advice [27]. Even though medical welfare systems differ from country to country, they proved important for the transition to motherhood to receive continuity of support at multiple times using a variety of methods, such as consultation by public health nurses and interaction with childcare support advisers and the community. This study suggests the importance of continuity of child-rearing support for transition to motherhood, not only in Japan, but also in the countries around the world with declining birthrates.

The mothers whose “property” of proactive involvement during pregnancy, childbirth, and child-rearing was strengthened by continuity of support incorporated the role of a mother into their own inner world, and re-shaped their identity as mothers through self-reflection and the socialization of child-rearing. They actively engaged in child-rearing through the new role of a mother and recognized their connection with society through the use of services and child-rearing, rather than taking it on alone. In order to promote the transition of mothers, it is necessary to encourage women to be proactively involved in pregnancy, childbirth, and child-rearing; to provide opportunities for mothers to reflect on their child-rearing objectively while maintaining their well-being; and to be involved so that they can reflect on their child-rearing in the community and have a sense of connection with the community. It is important to provide these forms of support throughout the child-rearing period, from pregnancy to postpartum, in order to enhance the readiness of mothers to raise their children, and to promote the re-shaping of their identities.

This study focused on the transition of mothers who raise their children while receiving continuity of child-rearing support. There were some limitations to this study. Since this is a model project, the number of participants was limited. Due to the small sample size, it is difficult to say that data saturation has been completely reached. All participants belonged to the same community, and therefore, there is a limit to the generalizability of the findings. In addition, all participants were Japanese. A wider range of sampling and a cross-cultural study should be conducted in the future to study cultural variations. In cases where the mother has some mental or social risk factors, it may be difficult to establish a relationship between the support provider and the mother, which complicates continuity of support. It is highly likely that the transition to motherhood proceeded smoothly for the participants of this study because they received continuity of support and were cooperative in the interviews, which limits the transferability of the study results.

## 5. Conclusions

This study clarified the importance of providing continuity of support for the transition to motherhood, such as encouraging women to be proactively involved in pregnancy, childbirth, and child-rearing; having opportunities to look at their child-rearing objectively while maintaining their well-being; and being involved in the community so that they can reflect on child-rearing in the community and have a sense of connection with the community. These results provide important evidence for child-rearing support in the future.

## Figures and Tables

**Table 1:** Characteristics of the participants (*n* = 13).

Characteristics	*n*
Age (Mean ± SD)	35.4 ± 4.8
Number of children = 1	7 (53.8%)
Number of children = 2	6 (46.2%)
Living with or near parents	3 (23.1%)
Employed	7 (53.8%)

## Data Availability

Not applicable.
